# Low host immune pressure may be associated with the development of hepatocellular carcinoma: a longitudinal analysis of complete genomes of the HBV 1762T, 1764A mutant

**DOI:** 10.3389/fonc.2023.1214423

**Published:** 2023-08-23

**Authors:** Zhi-Hua Jiang, Qin-Yan Chen, Hui-Hua Jia, Xue-Yan Wang, Lu-Juan Zhang, Xiao-Qian Huang, Tim J. Harrison, J. Brooks Jackson, Li Wu, Zhong-Liao Fang

**Affiliations:** ^1^ Guangxi Zhuang Autonomous Region Center for Disease Prevention and Control, Guangxi Key Laboratory for the Prevention and Control of Viral Hepatitis, Nanning, Guangxi, China; ^2^ School of Preclinical Medicine, Guangxi Medical University, Nanning, Guangxi, China; ^3^ Division of Medicine, University College London Medical School, London, United Kingdom; ^4^ Department of Pathology, Carver College of Medicine, University of Iowa, Iowa City, IA, United States; ^5^ Department of Microbiology and Immunology, Carver College of Medicine, University of Iowa, Iowa City, IA, United States

**Keywords:** hepatitis B virus, hepatocellular carcinoma, mutations, next-generation sequencing, quasispecies

## Abstract

**Background:**

It has been reported that hepatitis B virus (HBV) double mutations (A1762T, G1764A) are an aetiological factor of hepatocellular carcinoma (HCC). However, it is unclear who is prone to develop HCC, among those infected with the mutant. Exploring HBV quasispecies, which are strongly influenced by host immune pressure, may provide more information about the association of viral factors and HCC.

**Materials and methods:**

Nine HCC cases and 10 controls were selected from the Long An cohort. Serum samples were collected in 2004 and 2019 from subjects with HBV double mutations and the complete genome of HBV was amplified and sequenced using next-generation sequencing (NGS).

**Results:**

The Shannon entropy values increased from 2004 to 2019 in most cases and controls. There was no significant difference in mean intrahost quasispecies genetic distances between cases and controls. The change in the values of mean intrahost quasispecies genetic distances of the controls between 2004 and 2019 was significantly higher than that of the cases (P<0.05). The viral loads did not differ significantly between cases and controls in 2004(p=0.086) but differed at diagnosed in 2019 (p=0.009). Three mutations occurring with increasing frequency from 2004 to 2019 were identified in the HCC cases, including nt446 C→G, nt514 A→C and nt2857T→C. Their frequency differed significantly between the cases and controls (P<0.05).

**Conclusions:**

The change in the values of mean intrahost quasispecies genetic distances in HCC was smaller, suggesting that HBV in HCC cases may be subject to low host immune pressure. Increasing viral loads during long-term infection are associated with the development of HCC. The novel mutations may increase the risk for HCC.

## Introduction

Primary liver cancer is the seventh most frequently occurring cancer worldwide and the second most common cause of cancer mortality (1). It has a wide geographic variation in incidence. More than 80% of cases occur in sub-Saharan Africa and Eastern Asia (2). In Asia, liver cancer is the fifth most common cancer and the second most common cause of cancer-related death (3). In most countries, 75–90% of liver cancers are hepatocellular carcinoma (HCC) (4).

The risk factors of HCC are complex and vary geographically. In high-rate HCC areas, hepatitis B virus (HBV) and aflatoxin B1 (AFB1) are the dominant factors, whereas hepatitis C virus (HCV) and alcohol are more important factors in low- to medium-rate areas (4). Chronic infection by HBV is by far the most important risk factor for HCC in high-risk areas, including China and Africa (5). Up to 60-80% of HCCs are seropositive for markers of HBV infection (6). Male carriers of hepatitis B surface antigen (HBsAg) have a greater than 300-fold higher risk of developing HCC than antigen-negative controls (7). Universal immunization against hepatitis B already has had a favorable impact on the annual incidence of HCC in children and young adults (8, 9) confirming the causative role of the virus.

It has been proposed that some mutations in the genome of HBV may be involved in the development of HCC (10). G1896A, C1653T, T1753V, the basal core promoter (BCP) double mutations (A^1762^T, G^1764^A), and pre-S region deletions in the HBV genome have been reported to be associated with the development of HCC (11). The association of A1762T, G1764A double mutations and HCC has been confirmed by prospective cohort studies (12). However, it is unclear who is prone to develop HCC, among those infected with HBV with basal core promoter double mutations (1762T, 1764A).

HBV exists as a quasispecies (QS) in infected individuals (13). Various mutations may occur naturally in the HBV quasispecies during long-term infection (14). HBV quasispecies diversity, which is strongly influenced by host immune pressure, may increase the progression of fibrosis in chronic hepatitis B patients under immune selection (15). Therefore, exploring HBV quasispecies may provide more information about the relationship between viral factors and the development of HCC. Compared to Sanger DNA sequencing for the detection of HBV genetic diversity, next-generation sequencing (NGS) can simultaneously sequence a large number of viral genomes with high sensitivity and specificity (16). However, longitudinal data from NGS of complete HBV genomes is lacking. In this study, NGS technology was used to explore the long-term evolution of complete genome in the HBV quasispecies before the diagnosis of HCC, based on the Long An cohort (12).

## Materials and methods

### Study subjects and ethics statement

To determine the long-term evolution of HBV in HCC cases and control, a nested case-control study was carried out based on the Long An cohort (12), which was established in 2004. The cohort comprises 2,258 asymptomatic HBsAg carriers (ASC), then aged 30–55 years and living in the rural area of Long An county, Guangxi, China, including a group with BCP double mutations and a wild type BCP group. The study subjects were followed up for three years from 1st July, 2004. Each study subject completed a one-page questionnaire at their first visit, provided a serum sample every six months for the assessment of virological parameters and alpha fetal protein (AFP) concentrations, and was monitored for HCC by ultrasonography (US). Then, HCC cases were followed every year and collected blood samples where possible. This cohort was followed up in 2019 and serum samples were collected again.

All cases of HCC were diagnosed at the Long An people’s Hospital. The diagnosis was made by one or more of the following: (a) surgical biopsy; (b) elevated serum AFP (levels ≥400 µg/mL), excluding pregnancy, genital cancer, and other liver diseases including metastasis of tumors from other organs, plus clinical symptoms or one image (US or computed tomography, CT); (c) elevated serum AFP (levels <400 ng/mL), excluding pregnancy, genital cancer, and other liver diseases including metastasis of tumors from other organs, plus two images (US and CT) or one image (US or CT) and two positive HCC markers such as DCP, GGT II, AFU, CA19–9, etc.

The critical selection criterion is that both cases and controls were infected with HBV with BCP double mutations at baseline or later during follow up, so that the confounding effect of BCP double mutations could be controlled and other mutations associated with HCC could be identified. All samples were tested for anti-HCV was detected and those positive were excluded, to eliminate the confounding effect of HCV infection on the incidence of HCC. Informed consent in writing was obtained from the study subject. The study protocol conforms to the ethical guidelines of the 1975 Declaration of Helsinki and has been approved by the Guangxi Institutional Review Board (GXIRB2020-0021).

### Serological testing, measurement of serum viral loads, PCR for HBV genomic DNA, library preparation and next-generation sequencing, NGS data preprocessing, haplotype construction and diversity analysis and estimation of the intra-host HBV evolutionary rate

These methods have been reported previously (17). In brief, total DNA was extracted from 200 μL of each patient’s serum and full-length HBV genomic sequences (~3kb) were amplified and enriched, using the primer pair P1 and P2, according to the protocol of Günther et al. (18). If the mass of amplicon was insufficient, a second round of PCR was carried out using nested primers. All final products were confirmed by electrophoresis through 1% agarose gels. Next generation sequencing libraries were then prepared and sequenced on the Novaseq sequencer, with 150 bp PE reads in Delivectory Biosciences Inc. Company (Beijing, China). Quality control and preprocessing of each HBV sample’s raw reads was performed by fastp v0.20.1 (19). The clean reads were then mapped to a common reference sequence (accession no. X02763). After deduplication, a consensus HBV genome sequence was generated for each sample using Cliquesnv v1.5.3. All HBV consensus sequences were multi-aligned with references from HBVdb and several additional sequences. A maximum-likelihood tree was constructed with MEGA 7 (20) and the consensus sequence from each sample was genotyped.

### Shannon entropy and mutation analysis along the HBV genome

For each sample, the nucleotides at each position in the HBV genome were evaluated using the Samtools mpileup algorithm (21). The Shannon entropy of each site was calculated according to the nucleotide’s frequency and the differences between 2019 and 2004 were calculated for the paired samples. HBV SNV and Indels were obtained from the mpileup result and mutations related to HCC and ASC were investigated further. Mutations with a high frequency in year 2004 and 2019, or with an increasing frequency from 2004 to 2019 in the HCC group, or with a decrease in frequency from 2004 to 2019 in the ASC group, were considered to be associated with HCC. Mutations with a high frequency in year 2004 and 2019, or with a frequency increasing from 2004 to 2019 in the ASC group, or with a decrease in frequency from 2004 to 2019 in the HCC group, were considered to be protective. Only mutations which occurred in at least two cases in the same group were investigated further.

### HBV genotyping

**Figure 1:**
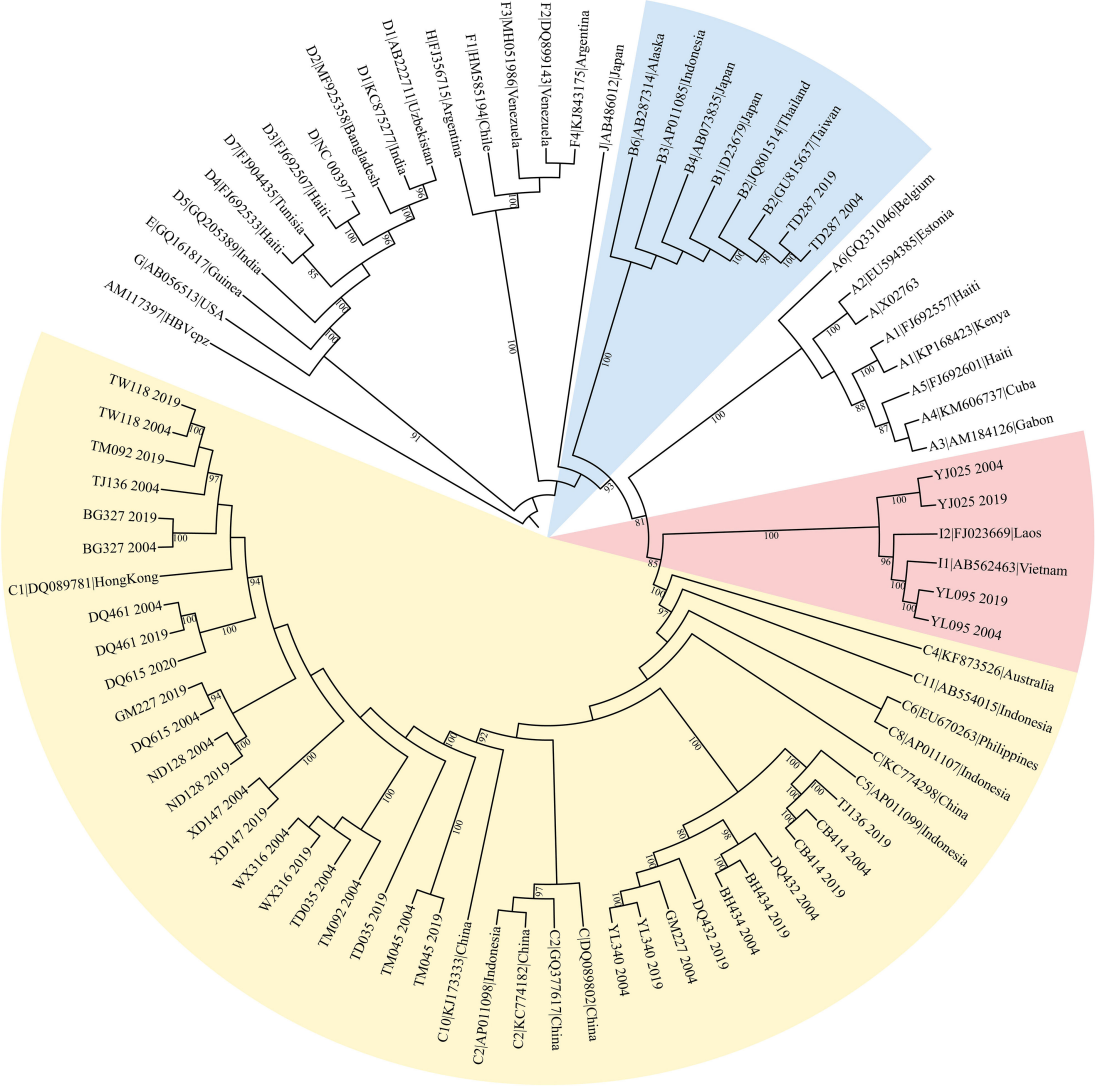
The maximum likelihood tree for genotyping the consensus sequences of 19 subjects. The yellow colored clade contains sequences from 18 genotype C subjects, BH434, WX316, etc. The pink colored clade contains sequences from 3 genotype I subjects, YJ025, HCC03 and YL095. The blue marked clade contains sequences from 1 genotype B subject, TD287.

HBV genotypes were determined using phylogenies reconstructed on the basis of the complete genome and preS/S regions of the viruses. The sequences were aligned to 45 HBV sequences of all known genotypes retrieved from GenBank using Clustal W and visually confirmed with the sequence editor BioEdit (22). The reference sequences are shown in **Figure 1**. Neighbor-Joining trees were reconstructed under the Kimura 2-parameter substitution model with the program MEGA (23). The reliability of clusters was evaluated using the interior branch test with 1000 replicates and internal nodes with over 75% support were considered reliable.

### Statistical methods

Statistical comparisons of the prevalence of HBV mutations between the case and control groups were performed using Pearson’s χ^2^ tests, McNemar’s test and Fisher’s exact test. The data of evolutionary rates are presented as median (range). Viral loads, genetic diversity, Sn value and evolutionary rates were compared between groups using the Mann-Whitney test. All P-values were two-tailed and P <0.05 was considered to be significant. All statistical analyses were performed using the SPSS software (ver.16.0; Chicago, IL, USA).

## Results

### General characteristics and genotypes

The original sample size was 13 cases and the paired controls. However, amplification of the complete HBV genome failed for some cases and controls. Eight cases with paired controls plus one HCC case and two asymptomatic controls that could not be paired were included. ALL were included in the analysis, making nine HCC cases and 10 ASC controls. Almost all (16/19) were infected with genotype C HBV (**Figure 1**). The proportion of that genotype in cases and controls is 88.9% (8/9) and 80% (8/10), respectively. There was no significant difference in the prevalence of genotype C between cases and controls (p=1). No genotype shifting was found during the 15 years. There is no significant difference in HBeAg positivity between cases and controls (p=1). The number of quasispecies of case group in 2004 and 2019 was 191and 205, respectively. And that of control group in 2004 and 2019 was 195 and 254, respectively (**Table 1**).

**Table 1:** HBV Quasispecies genetic distance and evolutionary rates for each study subject.

Subjects	Sex	Age#	Diagnosis	HBeAg§	Genotypes	Viral Loads (IU/ml)		No. Quasispecies		Genetic diversity			*S*n*		Evolutionary rates◇ (Median) (95% CI, lower- upper)
						2004	2019	2004	2019	2004	2019	2019 vs. 2004	2004	2019	
YJ025	F	50	ASC▽	-	I	3.70E+03	5.10E+03	26	30	0.005475	0.010905	0.011868	2.76	2.53	6.43E-04 (4.49E-04~9.64E-04)
TD035	M	40	HCC▼	–	C	9.50E+05	3.26E+05	3	28	0.000833	0.008839	0.019653	0.26	2.67	1.01E-03 (1.69E-04~2.02E-03)
BH434	M	37	ASC	–	C	9.05E+02	3.50E+04	50	39	0.004221	0.007448	0.019207	2.45	3.03	1.14E-03 (7.71E-04~1.77E-03)
WX316	M	32	ASC	+	C	1.00E+08	9.10E+05	3	30	0.001245	0.003951	0.007699	0.33	3.00	3.62E-04 (3.23E-07~8.39E-04)
TM092	M	41	HCC	–	C	1.83E+02	3.80E+03	13	14	0.01259	0.002488	0.022412	1.31	1.61	8.36E-04 (4.38E-04~1.37E-03)
ND128	F	35	HCC	–	C	8.20E+07	2.45E+05	7	13	0.000535	0.002006	0.00505	0.97	2.35	3.33E-04 (3.72E-05~7.48E-04)
YL340	F	35	ASC	+	C	3.51E+08	1.65E+03	5	18	0.002624	0.017719	0.018715	1.12	1.58	1.62E-04 (7.66E-08~5.91E-04)
TW118	M	36	HCC	–	C	5.20E+04	1.30E+06	31	29	0.004237	0.007409	0.00762	2.76	2.77	3.29E-04 (3.86E-05~7.34E-04)
TD287	M	32	ASC	–	B	7.20E+03	2.90E+04	9	29	0.012794	0.005082	0.016996	0.83	2.68	7.51E-04 (3.91E-04~1.25E-03)
TM045	F	45	HCC	–	C	14669	8.01E+05	28	29	0.006523	0.00828	0.0133	2.84	2.96	3.10E-04 (1.42E-04~5.84E-04)
CB414	F	38	ASC	–	C	3.40E+03	3.84E+02	35	34	0.024666	0.014467	0.025496	2.82	2.61	7.80E-04 (5.49E-04~1.11E-03)
XD147	M	38	HCC	–	C	2.60E+04	2.00E+04	5	20	0.001251	0.00337	0.006036	1.13	2.67	3.85E-04 (9.52E-05~7.08E-04)
DQ432	M	31	ASC	–	C	1.71E+02	3.47E+00	8	7	0.0434	0.00563	0.05734	1.20	1.49	7.68E-04 (5.53E-05~1.74E-03)
BG327	F	45	HCC	–	C	1.20E+05	6.10E+06	33	16	0.007353	0.004572	0.010565	2.84	1.86	4.22E-04 (1.91E-04~8.05E-04)
DQ461	F	42	ASC	–	C	5.90E+03	4.90E+03	29	30	0.003211	0.006808	0.010428	2.93	2.84	6.89E-04 (4.19E-04~1.09E-03)
TJ136	F	36	ASC	–	C	78.1	2.10E+00	2	10	0.000622	0.005654	0.071849	0.10	1.08	4.38E-03 (1.13E-03~5.96E-03)
DQ615	M	35	HCC	–	C	9.80E+06	5.30E+07	37	37	0.014659	0.01463	0.016726	2.76	2.76	4.72E-04 (2.98E-04~6.71E-04)
YL095	F	45	HCC	–	I	3.56E+07	1.61E+06	34	19	0.004714	0.004295	0.008623	3.01	2.61	4.41E-04 (2.04E-04~7.89E-04)
GM227	F	45	ASC	–	C	1.13E+04	4.06E+04	28	27	0.009673	0.006324	0.069729	2.72	2.76	4.13E-03 (2.76E-03~5.40E-03)

The age in 2004. ▽ASC: Asymptomatic HBsAg carriers. ▼HCC: Hepatocellular carcinoma. §: Detected in 2019. *Sn: Shannon entropy. **◇**:Substitutions per site per year.

### Quasispecies Shannon entropy and intrahost genetic distance

**Figure 2:**
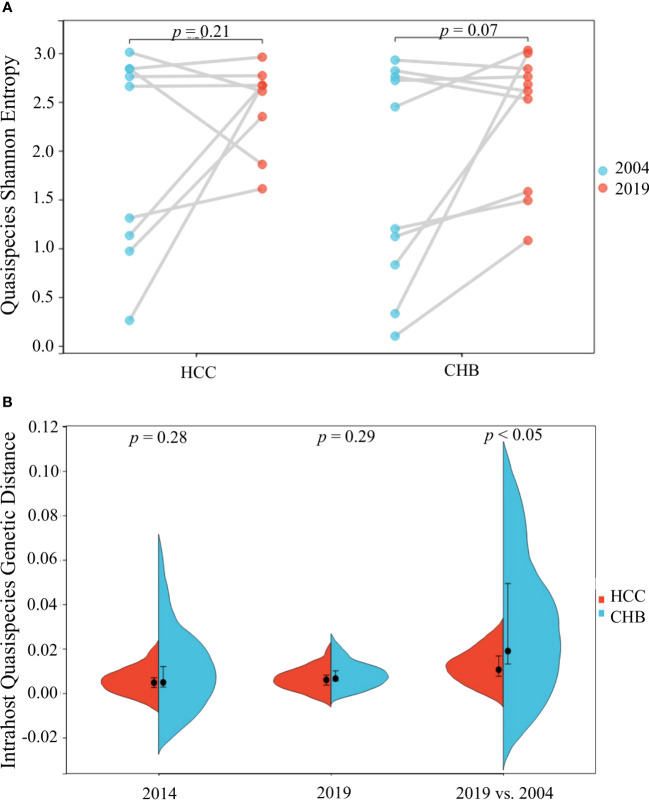
HBV intra-host viral quasispecies Shannon entropy differences between 2004 and 2019 **(A)** and genetic diversity distribution **(B)** of the 9 HCC and 10 CHB subjects.

The quasispecies Shannon entropies of each subject in the HCC and control groups are shown in **Table 1** and **Figure 2A**. The Sn values of the HCC case group (1.9867 in 2004 and 2.4733 in 2019) and control group (1.726 in 2004 and 2.36 in 2019) did not differ significantly (p=0.368 in 2004, p=1.000 in 2019, respectively). However, most of the values increased from 2004 to 2019 in both groups, indicating that the quasispecies structure increased in complexity over time. Most of the mean intrahost quasispecies genetic distances in the HCC cases and controls were less than 1% in 2004 and 2019 and there was no significant difference between the two groups (p=0.683 in 2004; p=0.253 in 2019, respectively), as shown in **Table 1** and **Figure 2B**. However, most of the mean intrahost quasispecies genetic distances of the controls between 2004 and 2019 were greater than 1%. The values were significantly higher than the HCC cases (*p*<0.05), suggesting that the viruses in HCC cases may be subject to lower immune pressure from the host.

### Shannon entropy change along the genome

**Figure 3:**
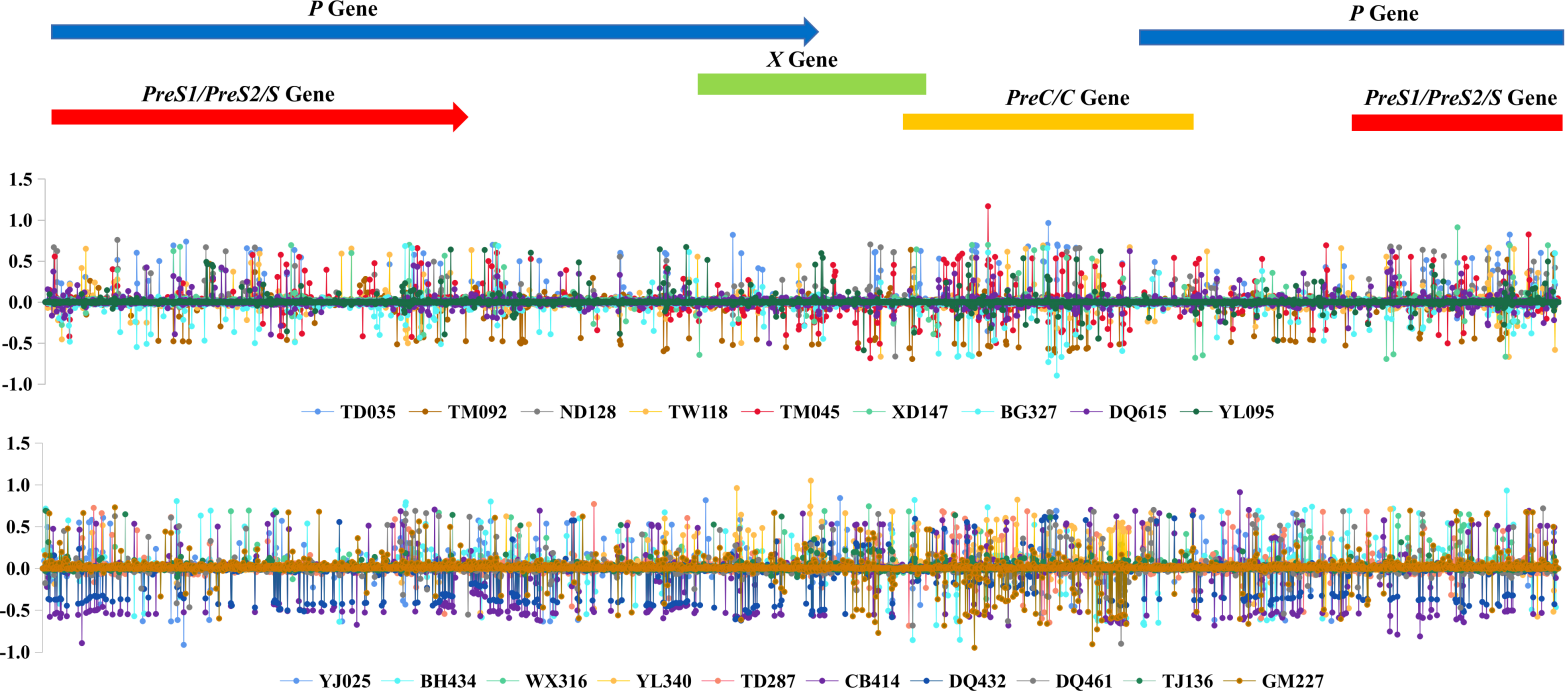
HBV intra-host viral quasispecies’ 2019 and 2004 Shannon entropy differences, according to the genomic location.

The Shannon entropy values of each site along the HBV genome were calculated for each sample, and the difference between 2004 and 2019 was further calculated for each subject, if available, as shown in **Figure 3**. The plot suggested that the viral nucleotide distribution tends to change much more in the PreC/C and PreS1/PreS2/S regions in HCC patients over time, while no such outcome was seen in the controls.

The details of each subject’s intrahost viral evolutionary rates are summarized in **Table 1**. The highest and lowest evolutionary rates in the HCC group are 1.01E-03 and 3E-04. The highest and lowest evolutionary rates in the control group are 4E-03 and 2E-04. The difference in the median value of the substitution rate between the case and control groups is not significant (p=0.102).

### Changes in viral loads

The details of each subject’s viral loads in 2004 and 2019 are summarized in **Table 1**. The viral loads of HCC cases in 2019 were not significantly different from 2004 (p=0.757). Similar results are seen for the control group. The viral loads in 2019 did not differ significantly from 2004 (p=0.0.821). The viral loads of HCC cases in 2004 did not differ significantly from the control group in 2004 (p=0.086). However, the viral loads of HCC cases in 2019 were significantly different from those of the control group in 2019 (p=0.009), suggesting that increasing viral loads are associated with the development of HCC.

### Mutations in the entire HBV genome characteristic of HCC

The prevalences of the C1653T mutation in the case and control group in 2004 were 44.4% (4/9) and 10% (1/10), respectively, and the values in 2019 were 55.4% (5/9) and 40% (4/10), respectively. The prevalences of the T1753V mutation in the case and control groups in 2004 were 33.3% (3/9) and 30% (3/10), respectively, and the values in 2019 were 33.3% (3/9) and 40% (4/10), respectively. The prevalences of PreS1/S2 deletions or PreS2 start mutations in the case and control groups in 2004 were 77.8% (7/9) and 60% (6/10), respectively, and the values in 2019 were 66.7% (6/9) and 70% (7/10), respectively. However, none of these differences is significant, suggesting that the C1653T mutation, the T1753V mutation and the PreS1/S2 deletion or PreS2 start mutations are not associated with the development of HCC (**Table 2**).

**Table 2:** Mutations suggested to be associated with the development of HCC.

Subjects	Years	No. Quasispecies	C1653T	T1753V	PreS2 start	PreS1/S2 Deletion (nt*)
YJ025	2004	26	0	0	11(ATA)	
	2019	30	0	0	20(ATA), 5(ACA),4(GCA)	1 (1792–1839)
TD035	2004	3	0	0	N	1 (1857–1862,1864–1878,1881–1889)
	2019	28	0	0	N	
BH434	2004	50	0	0	N	1 (1793–1795),1(1843-1849)
	2019	39	0	0	N	
WX316	2004	3	0	0	0	
	2019	30	0	30-C	N	2(317-319,2848-2853,2859-2876)
TM092	2004	13	0	7C	N	13(1798-1806)
	2019	14	0	14C	N	14(3215-56)
ND128	2004	7	0	0	N	1(1754-1763),3(2849-2865)
	2019	13	0	0	N	
YL340	2004	5	0	0	1GTG,1ACG	1(1667-1676,1763-1780,3215-2)
	2019	18	11	0	16ATA	
TW118	2004	31	0	0	5ATA,3ACA	1(3029-3077)
	2019	29	29	0	9ACA	
TD287	2004	9	0	0	N	
	2019	29	0	0	N	
TM045	2004	28	9	5G,19C	N	5(1316-1318,2848-2867),3(2848-2867),1(317-319),5(3128-3159)
	2019	29	1	29C	N	18(1316-1318,2848-2867),1(1819-1826)
CB414	2004	35	0	33C	N	5(3215-2,1845-1847)
	2019	34	0	29C	4ACG,7ATT	3(3215-2,573-575),1(3215-2,1368-1370) 1(3215-2,1817-1820)
XD147	2004	5	5	0	0	
	2019	20	20	0	N	2(2848-2865),1(2073-2081,2848-2876)
DQ432	2004	8	6	0	4ATA	1(552-559),1(1794-1805)
	2019	7	7	0	N	
BG327	2004	33	33	0	N	1(1841-1846,1849-1859),1853-1851), 1(2119-2123),1(2894-2934)
	2019	16	16	0	13ATA	1(1417-1423)
DQ461	2004	29	0	29C	N	
	2019	30	3T	30C	N	2(1849-1859)
TJ136	2004	2	0	0	N	
	2019	10	10A	2	10ACG	
DQ615	2004	37	11T	0	14GTG	2(2051-2057),1(2127-2159),1(2149-2151), 1(2860-2867),1(2848-2864)
	2019	37	21T	0	11GTG	4(2860-2867),3(2051-2057),2(2952-2973)
YL095	2004	34	0	2A,32C	N	
	2019	19	0	19C	N	
GM227	2004	28	0	9C	N	2(2620-2622),1(1838-1850),1(1976-1978)
	2019	27	0	0	24GTG	10(1820-1822)

*: Nucleotide.

In this study, four novel mutations were identified with increasing frequency from 2004 to 2019 in HCC group, while the frequency of these mutations did not increase or decrease from 2004 to 2019 in the control group. The frequency of these mutations differed significantly between the case group and control group. These mutations, nt446 C→G (P=0.033) and nt514 A→C (P=0.033) in the S open reading frame (ORF), nt2170 T→C (P=0.033) in the C ORF and nt2857T→C (P=0.033) in the PreS2 region, seem to be associated with the development of HCC (**Table 3**).

**Table 3:** Newly identified mutations associated with higher risk for HCC.

nt^$^	HCC^▼^	ASC^▽^
2170T→C	TD035 (0.5923^#^/0.0009*), ND128 (0.3237/0.0024), XD147 (0.225/0), DQ615 (0.5959/0.2774).	0
514A→C	TD035 (0.1072/0.00060), TM092 (0.9995/0.181), DQ615 (0.1223/0.0019), YL095 (0.9622/0.7921), XD147 (0.9898/0.999).	0
2857T→C	TD035 (0.1305/0.0006), TM092 (0.999/0.0022), ND128 (0.5617/0.0034), DQ615 (0.2671/0.0206), TW118 (0.9993/0.9794).	CB414 (0.0059/0.0645).
446C→G	TD035 (0.3537/0.0002), ND128 (0.3925/0.0005), BG327 (0.9949/0.7789), DQ615 (0.3539/0.1959).	DQ461 (0.999/0.9991).
162A→G	DQ615 (0.714/0.4135), TD035 (0.9952/0.9979), TM092 (0.9985/0.9981), ND128 (0.9971/0.9979), TW118 (0.995/0.9992), TM045 (0.9939/0.9929), XD14 7(0.9986/0.9988), BG327 (0.9977/0.9989).	TJ136 (0.0028/0.995), WX316 (0.997/0.9979), DQ461 (0.9989/0.9895).
3051C→T	TD035 (0.9988/0.999), TM092 (0.9965/0.9978, ND128 (1/0.9966), TW118 (0.9986/0.9993), TM045 (0.9976/0.9974), XD147 (0.9972/0.9991), BG327 (0.9988/0.9951), DQ615 (0.9966/0.9929).	TJ136 (0.0016/0.9985), WX316(0.9992/0.999), DQ461 (0.9843/0.9965).
633A→G	TD035 (0.9967/0.9981), TM092 (0.9988/0.999), ND128 (1/0.9987), TW118 (0.9986/0.9962), TM045 (0.9992/0.9947); XD147 (0.9981/0.9991); BG327 (0.9981/0.9949), DQ615 (0.9984/0.9952).	TJ136 (0.0021/0.9978), WX316 (0.9982/0.9985), DQ461 (0.9993/0.9986).
2567C→T	TD035 (0.9994/0.9991), TM092 (0.9965/0.9977), ND128(0.9971/0.9976), TW118(0.9987/0.9995), TM045 (0.9985/0.9964), XD147 (0.9985/0.9991); BG327(0.999/0.9918), DQ615(0.9889/0.9831).	DQ461 (0.2568/0.9932), TJ136 (0.0005/0.9993), WX316(0.9995/0.9993).
1155C→T	TD035 (0.9974/0.9986), TM092 (0.9978/0.9973), ND128 (0.9981/0.9977), TW118 (0.9993/0.9996), TM045 (0.999/0.9964), XD14 7(0.9922/0.9998), BG327 (0.9979/0.9968), DQ615(0.997/0.9919).	TJ136 (0.0008/0.9988), WX316 (0.9991/0.9988), DQ461 (0.9976/0.9975).
3026C→T	TD035 (0.9996/0.9993), TM092 (0.9978/0.9978), ND128 (0.9972/0.9986), TW118 (0.9996/0.9993), TM045 (0.9928/0.9969), XD147 (0.9958/0.9994), BG327 (0.9993/0.997), DQ615 (0.9696/0.992).	BH434 (0.7899/0.9989), WX316 (0.6378/0.9988), DQ432 (0.001/0.8297), TJ136 (0.0018/0.9985), DQ461 (0.9976/0.9976).

**^$^:** Nucleotide. ^▼^HCC: Hepatocellular carcinoma. ^▽^ASC: Asymptomatic HBsAg carriers. ^#^: Frequency of mutation in 2019; ^*^: Frequency of mutation in 2004.

nt514 A→C is a synonymous mutation in the S ORF but is a missense mutation in the overlapping polymerase ORF, affecting codon 129 (methionine to leucine). nt2170 T→C is a synonymous mutation in the C ORF. nt446 C→G is a missense mutation, causing the change of leucine to valine at codon 98 of the S ORF and serine to cysteine at codon 106 of the overlapping polymerase ORF. nt2857T→C is a missense mutation, resulting in the change of tryptophan to arginine at codon 4 of the PreS2 domain and leucine to serine at codon 184 of the overlapping polymerase ORF (**Table 3**).

## Discussion

The major finding of this study is that the quasispecies structure of HBV with BCP double mutations increased in complexity over time. The change in the values of mean intrahost quasispecies genetic distances of the controls between 2004 and 2019 were significantly higher than the HCC cases, suggesting that HBV in the HCC cases may be subject to lower host immune pressure. Nucleotides in the PreC/C and PreS1/PreS2/S ORFs tended to change much more frequently over time in the HCC patients than in the asymptomatic carriers. Four novel mutations were identified that are associated with the development of HCC; three of them are missense mutations that affect codons in the PreS2/S and polymerase ORFs. Increasing viral loads during long-term infection also are associated with the development of HCC. The strength of this study is that the data were derived from the long-term evolution of HBV quasispecies before the diagnosis of HCC, which may provide information about the change of intrahost quasispecies genetic distances and the causative role of the mutations. The weakness of this study is that the sample sizes were insufficient for multivariable logistic regression analysis.

It has been reported that the characteristics of viral quasispecies are associated with the exacerbation of liver fibrosis progression and the development of liver cancer (15, 24). NGS provides higher sensitivity and specificity for detecting quasispecies than Sanger DNA sequencing (16). Therefore, this technique has been used to search for quasispecies and the mutations associated with the development of HCC and has produced some interesting results (25–28). All of these studies investigated the PreS region only, except for Chang’s group, who investigated the complete genome of HBV from HCC patients and non-HCC controls and found 41 novel HCC-associated SNVs and preS deletions that involved HBV ORFs and regulatory elements (26). Unfortunately, that was a cross-sectional study and could not provide information about the change of intrahost quasispecies and mutations before the diagnosis of HCC. Therefore, the results obtained in this study should be more reliable, because both HCC cases and controls were selected from a prospective cohort and serum was available prior to HCC diagnosis.

Although HBV infection has long been established as a major cause of HCC, the mechanisms of oncogenesis remain obscure (29). Nonetheless, the quasispecies associated with tumor development recently have become a major focus for research. The characteristics of viral quasispecies in the PreS region have been reported to be associated with the development of HCC. HBV polymerase sequences may contain vital HBV quasispecies features which may be used to predict HCC (30). Quasispecies diversity was found to be strongly influenced by host immune pressures (14). The findings here that low host immune pressure may be associated with the development of hepatocellular carcinoma is important for understanding the mechanisms of oncogenesis of HBV. All individuals infected with the HBV 1762T 1764A mutant, should be screened regularly for HCC among those so as to detect the tumor at an early stage, because those exerting low immune pressure on the virus may be at particularly high risk of HCC.

It has been reported that there is a subtle relaxation of selection pressure on the HBV core gene in subjects with HBeAg-negative chronic hepatitis B. This may be attributable to impaired antiviral immunity, and could contribute to the high levels of viral replication (31). In this study, all HCC cases infected with HBV with basal core promoter double mutations (A1762T, G1764A) were negative for HBeAg and their viral loads are significantly higher than those of the controls. High HBV viral loads are a risk factor of HCC (32). High rates of replication of HBV may lead directly to increased numbers of chromosomal integration events and, thus, to HCC (33). Therefore, it is reasonable to postulate that the antiviral immunity of the HCC patients was impaired, resulting in low immune pressure on the replicating virus. If so, this may provide clues as to who is prone to develop HCC, among those infected with HBV with basal core promoter double mutations (A1762T, G1764A).

HBV mutations, in the preS or PreC and/or core promoter regions, have been recognized to be significantly associated with HCC (34). This conclusion was further supported by this study. Some other studies have suggested that the core promoter mutations C1653T and T1753V also are associated with the occurrence of severe hepatitis B and the development of HCC (35). However, this suggestion was not supported by the current or previous results (12). The occurrence of deletions in the PreS region was prominently more common in HCC patients than ASC patients (36, 37). It surprises us that the current results do not support the association of PreS deletions and the development of HCC, although both studies are based on the same cohort. This may be attributable to the small sample size. Although the four novel mutations may increase the risk of HCC, there was no significant difference in the prevalence of these mutations between HCC patients and controls. These mutations may not be factors leading to HCC, but only co-factors. However, these need to be investigated further.

A prospective study reported that HBV viremia, except perhaps at extremely low levels, is associated with an increased risk for HCC; those with high viral loads of 3.8 x 10^4^ virions/ml at entry are at increased risk of HCC (38). In this study, no significant difference was found in viral loads between the cases and control groups at entry, although the difference became significant when HCC was diagnosed. Therefore, evaluation of the risk for HCC based on a single measurement of viral load may not be sufficient.

Reflecting virus–host interplay, increased HBV quasispecies complexity and diversity in the pre-S region was found to be associated with the development of HCC (25). These findings were not confirmed by this study. In contrast, the mean intrahost quasispecies genetic distances of controls between 2004 and 2019 were found to be significantly higher than those of the HCC cases. This may be attributable to these results having been derived from complete genome sequences.

## Conclusions

The quasispecies structure of HBV with BCP double mutations may increase in complexity over time. The change in the values of mean intrahost quasispecies genetic distances of HCC cases were significantly lower than the controls, suggesting that HBV in HCC cases may be subject to lower host immune pressure and result in high replication of the virus during long-term infection. The findings may provide a novel clue to understand the mechanisms of oncogenesis of the HBV 1762T,1764A mutant.

## Data availability statement

The original contributions presented in the study are included in the article/GenBank. Further inquiries can be directed to the corresponding author.

## Ethics statement

The studies involving humans were approved by The Guangxi Institutional Review Board (GXIRB2020-0021). The studies were conducted in accordance with the local legislation and institutional requirements. The participants provided their written informed consent to participate in this study.

## Author contributions

Conceptualization: LW, Z-LF, TH, JJ. Data curation: Z-HJ, Q-YC, H-HJ, X-YW. Formal analysis: Z-HJ, X-YW. Investigation: Z-HJ, Q-YC, H-HJ, L-JZ, X-QH. Methodology: Z-HJ, X-YW. Supervision: LW, Z-LF. Writing- original draft: Z-LF, Z-HJ. Writing-review & editing: LW, Z-LF, TH, JJ. All authors read and approved the final manuscript. All authors met the ICMJE authorship criteria.
